# Molecular Dynamics of Lithium Ion Transport in a Model Solid Electrolyte Interphase

**DOI:** 10.1038/s41598-018-28869-x

**Published:** 2018-07-16

**Authors:** Ajay Muralidharan, Mangesh I. Chaudhari, Lawrence R. Pratt, Susan B. Rempe

**Affiliations:** 1 Tulane University, Department of Chemical and Biomolecular Engineering, New Orleans, 70118 USA; 2 Sandia National Laboratories, Center for Biological and Engineering Sciences, Albuqueruque, 87185 USA

## Abstract

Li^+^ transport within a solid electrolyte interphase (SEI) in lithium ion batteries has challenged molecular dynamics (MD) studies due to limited compositional control of that layer. In recent years, experiments and *ab initio* simulations have identified dilithium ethylene dicarbonate (Li_2_EDC) as the dominant component of SEI layers. Here, we adopt a parameterized, non-polarizable MD force field for Li_2_EDC to study transport characteristics of Li^+^ in this model SEI layer at moderate temperatures over long times. The observed correlations are consistent with recent MD results using a polarizable force field, suggesting that this non-polarizable model is effective for our purposes of investigating Li^+^ dynamics. Mean-squared displacements distinguish three distinct Li^+^ transport regimes in EDC — ballistic, trapping, and diffusive. Compared to liquid ethylene carbonate (EC), the nanosecond trapping times in EDC are significantly longer and naturally decrease at higher temperatures. New materials developed for fast-charging Li-ion batteries should have a smaller trapping region. The analyses implemented in this paper can be used for testing transport of Li^+^ ion in novel battery materials. Non-Gaussian features of van Hove self -correlation functions for Li^+^ in EDC, along with the mean-squared displacements, are consistent in describing EDC as a glassy material compared with liquid EC. Vibrational modes of Li^+^ ion, identified by MD, characterize the trapping and are further validated by electronic structure calculations. Some of this work appeared in an extended abstract and has been reproduced with permission from ECS Transactions, 77, 1155–1162 (2017). Copyright 2017, Electrochemical Society, INC.

## Introduction

During charging and discharging cycles of lithium ion batteries, a solid electrolyte interphase (SEI) layer forms on the negative electrode due to decomposition of solvents like ethylene carbonate (EC). The SEI layer is a complex organic material and its composition is not operationally set^1^. Nevertheless, dilithium ethylene dicarbonate (Li_2_EDC) has been identified experimentally as the primary component of the outer part of the SEI layer^2–5^.

*Ab initio* molecular dynamics (AIMD) simulations, electronic structure calculations^6–8^ and reactive force field simulations^9^ on the decomposition of ethylene carbonate (EC) on anode surfaces concur with those experimental results. Experimental observations also show that the SEI layer protects the electrode from further decomposition by blocking electron transport while simultaneously allowing transport of Li^+^ ions between the electrode and electrolyte solution. A better understanding of the transport mechanism of Li^+^ in EDC may lead to modified SEI layers with improved lithium ion battery performance.

Molecular dynamics (MD) studies on model SEI layers carried out over long time-scales may shed new light into the mechanism of transport of Li^+^ ions within the SEI layer. Borodin, *et al*.^10–12^, performed MD calculations using a specialized polarizable force field to obtain transport properties of Li^+^ ion in a model SEI layer composed of ordered and amorphous Li_2_EDC. Since polarizable force fields are not readily available in standard molecular dynamics packages, we have instead identified non-polarizable force field parameters^13^ for simulation of the Li_2_EDC model of the SEI layer. The microsecond time-scales studied here, longer than earlier work^10^, provide additional insight into structural and transport properties of Li^+^ ions in this model SEI.

## Results and Discussion

**Figure 1:**
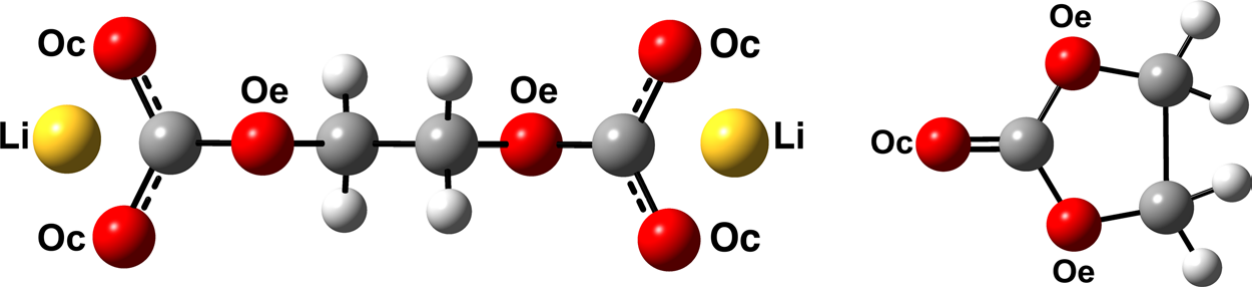
Chemical structure of Li_2_EDC (left) and EC (right) molecules. Carbon atoms are colored gray, hydrogens white, carbonyl (Oc) or ether (Oe) oxygen red, and Li^+^ ions yellow. (Redrawn with permission from *ECS Transactions* 77, 1155–1162 (2017))^13^.

We summarize the structural and transport properties from MD studies of a model SEI layer with 256 Li_2_EDC moieties (Fig. 1, redrawn from ref.^13^). In addition, simulations of a dilute Li^+^ ion in EC (single Li^+^ ion solvated by 249 EC) provide a perspective for comparison.

### Structural data

**Figure 2:**
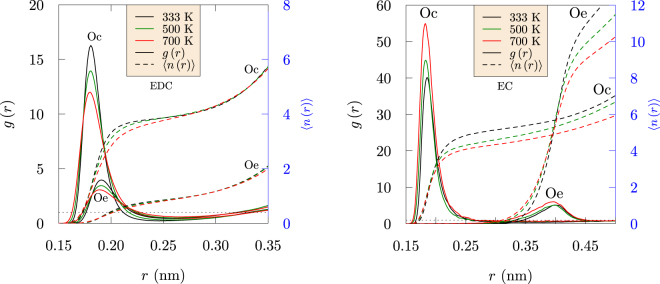
Radial distribution functions, *g*(*r*) and running coordination number, $\langle n(r)\rangle =4\pi \rho {\int }_{0}^{r}g(x){x}^{2}dx$, for Oc-Li^+^ and Oe-Li^+^ at various temperatures. For EDC (left), occupancy of the first solvation shell does not depend on the temperature, even though the peak height diminishes with increasing temperature. For liquid EC solvent (right), almost one additional Oc atom interacts with Li^+^ at lower temperature. (Redrawn with permission from *ECS Transactions* 77, 1155–1162 (2017))^13^.

The radial distribution functions (rdfs) and running coordination numbers (Fig. 2, redrawn from ref.^13^), involving Li^+^, carbonyl oxygen (Oc) and ether oxygen (Oe) of EDC and EC are compared at several temperatures. For EDC, the rdfs become less structured with increasing temperature, but the peak positions and overall coordination numbers of the first peak change only slightly. This structural robustness suggests an amorphous glassy matrix for the SEI. These results compare well with recent polarizable force field results^10,14^, supporting the applicability of the present non-polarizable force field for these structural characteristics. In EDC, the peak position for the Li-Oe distribution is shifted to a slightly larger value than Li-Oc because the charge on Oe is 40% smaller than that of Oc.

In the case of EC, the peak height increases with temperature and the peak position of the Li-Oe is farther out due to strong interactions between Li^+^ and Oc. The structure around Li^+^ changes significantly with temperature, as highlighted by the running coordination number. In contrast to glassy EDC, almost one additional carbonyl oxygen (Oc) atom interacts with Li^+^ at lower temperature for EC solvent.

### Mean-squared displacements of Li^+^

**Figure 3:**
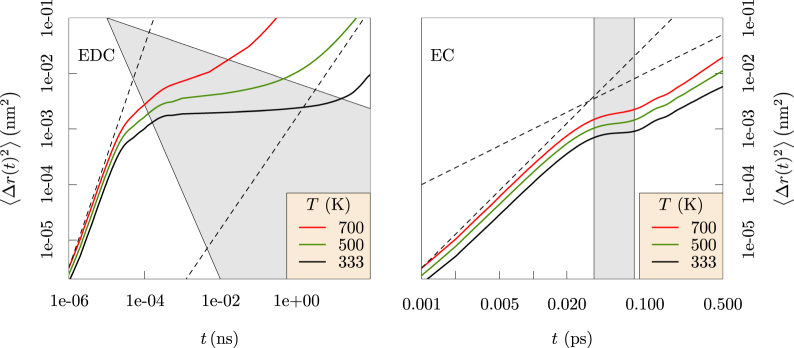
Mean-squared displacements, $\langle {\rm{\Delta }}r{(t)}^{2}\rangle$, measured for Li^+^ in EDC (left) and EC (right). The behavior in EDC at intermediate timescales 0.001< *t* < 1 (ns) demonstrates trapping of the Li^+^ ion. Ballistic motion $(\langle {\rm{\Delta }}r{(t)}^{2}\rangle \propto {t}^{2})$ is evident at short timescales, while diffusive motion $(\langle {\rm{\Delta }}r{(t)}^{2}\rangle \propto {t}^{1})$ appears at long timescales in both EDC and EC solvents. Dashed lines with slope 1 and 2 (log-scale) are provided as visual cues. At high *T*, the trapping regime (shaded region) diminishes and the EDC matrix behaves more like liquid EC. Note that the time scales differ dramatically between the two systems. (Redrawn with permission from *ECS Transactions* 77, 1155–1162 (2017))^13^.

The mean-squared displacements (MSD, Fig. 3, redrawn from ref.^13^) of Li^+^ ion in EDC and liquid EC distinguish three distinct dynamical regimes: ballistic at short times, trapping at intermediate times, and diffusive at long times. Trapping of Li^+^ in EDC is qualitatively different than in liquid EC, and the trapping times in EDC diminish as temperature increases and the glassy EDC matrix softens. Diffusivities of Li^+^ are extracted from the slope of the diffusive regime of MSD and then extrapolated to low temperatures using an Arrhenius fit (see Supplementary Information). The diffusivity of Li^+^ in EDC at 333 K is 10^−12^ cm^2^/s, which is in agreement with Borodin *et. al*.^10^. The conductivity of Li_2_ EDC obtained from the Nernst-Einstein equation (Supplementary Information) is 4.5 × 10^−9^ S/cm, which is also in agreement with experiment^10^.

### Time correlation functions for Li^+^ transport

The van Hove time correlation function

1 $$G(r,t)=\frac{1}{N}\langle \sum _{i=1}^{N}\sum _{j=1}^{N}\delta ({\bf{r}}+{{\bf{r}}}_{j}\mathrm{(0)}-{{\bf{r}}}_{i}(t))\rangle $$

for Li^+^ ion describes the probability of finding, at time *t*, a Li^+^ ion displaced by *r* from its initial position. This van Hove function can be split into *self* and *distinct* parts, *G*(*r*, *t*) = *G*_s_(*r*, *t*) + *G*_d_(*r*, *t*), with the latter taking the form

2 $${G}_{{\rm{d}}}(r,t)=\frac{1}{N}\langle \sum _{i\ne j}^{N}\delta ({\bf{r}}+{{\bf{r}}}_{j}(0)-{{\bf{r}}}_{i}(t))\rangle .$$

At *t* = 0, the van Hove function reduces to the static pair correlation function,

3 $$G(r,\,\mathrm{0)}=\delta ({\bf{r}})+\rho g(r\mathrm{).}$$

The *self* part of the van Hove function provides a jump probability, and the natural initial approximation is the Gaussian model,

4 $${G}_{{\rm{s}}}(r,t)={[\frac{3}{2\pi \langle {\rm{\Delta }}r{(t)}^{2}\rangle }]}^{\mathrm{3/2}}\times \exp [-(\frac{3{r}^{2}}{2\langle {\rm{\Delta }}r{(t)}^{2}\rangle })]\mathrm{.}$$

**Figure 4:**
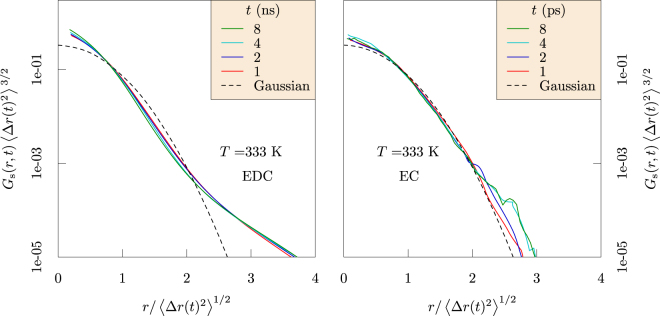
Dimensionally-scaled *G*_*s*_(*r*, *t*) for Li^+^ ions as it depends on displacement for increasing times in EDC (left) and EC (right). Though the Gaussian model (dashed curve) is reliable in EC solvent, probability is depleted near the trap boundaries, $r > {\langle {\rm{\Delta }}r{(t)}^{2}\rangle }^{\mathrm{1/2}}$, and replaced at shorter and longer distances for EDC. Note that these correlations decay in a few ps for EC, but require ns for EDC. (Redrawn with permission from *ECS Transactions* 77, 1155–1162 (2017))^13^.

For fluids like EC, this Gaussian behavior should be reliable. In contrast, *G*_s_(*r*, *t*) in the trapping regime of EDC indicates (Fig. 4, redrawn from ref.^13^) depletion of probability near the trap boundaries, $r > {\langle {\rm{\Delta }}r{(t)}^{2}\rangle }^{\mathrm{1/2}}$, and replacement of that probability at shorter and longer distances. Deviation from the Gaussian behavior can be characterized by the non-Gaussian parameter^15^

5 $$\alpha (t)=\frac{3\langle {\rm{\Delta }}r{(t)}^{4}\rangle }{5{\langle {\rm{\Delta }}r{(t)}^{2}\rangle }^{2}}-1.$$

**Figure 5:**
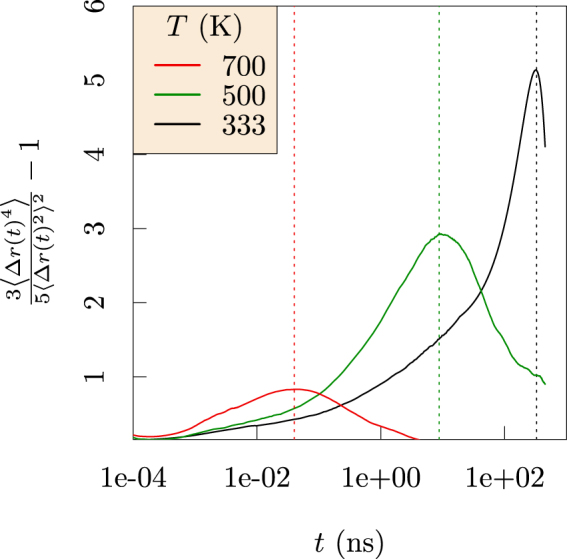
Non-Gaussian parameter calculated for Li^+^ in EDC at several temperatures. Vertical lines are drawn at the maximum value of *α*(*t*). The non-zero value and inverse temperature dependence of *α*(*t*) attests to the glassy behavior of EDC, which becomes more fluid-like at higher temperature.

**Figure 6:**
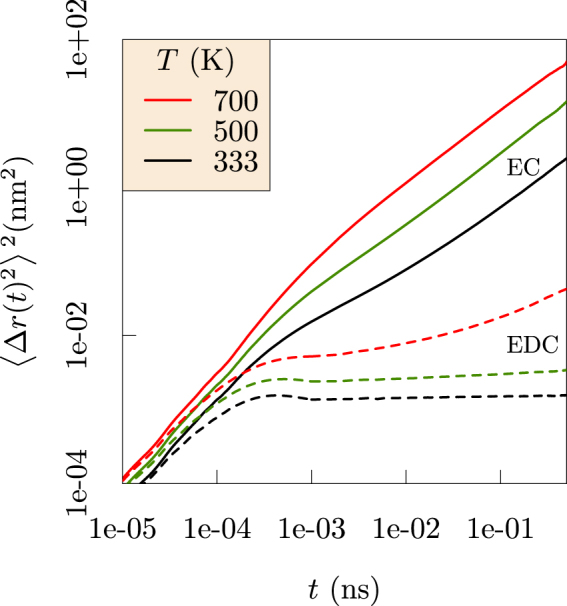
Comparison between mean-squared displacements of carbonyl carbon of EDC and EC. The flat MSD at larger time-scales reflects the glassy nature of the EDC matrix.

The van Hove self-correlation function is accurately Gaussian for liquid EC, hence *α*(*t*) = 0. In contrast, *α*(*t*) has non-zero values for glassy EDC (Fig. 5). For EDC, *α*(*t*) has a maximum that decreases with increasing temperature. The mean-squared displacements of the carbonyl carbons of EDC and EC molecules (Fig. 6) further verify the sluggish diffusion of EDC relative to EC. In the case of EDC, the mean-squared displacement curves are relatively flat for all temperatures, indicating little diffusion of the EDC matrix.

**Figure 7:**
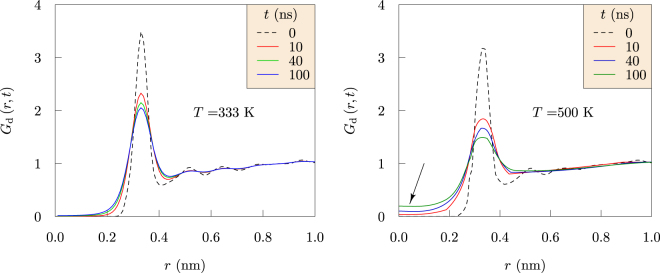
The Li-Li radial distribution function in EDC at *t* = 0 (dashed) and the corresponding *distinct* part, *G*_d_(*r*, *t*), of the van Hove function within the Vineyard approximation. The non-zero population in the core region surrounding *r* ≈ 0 (right panel) describes correlation of Li^+^ jumps, *i.e*., refilling a hole left by a Li^+^ ion with a neighboring Li^+^ ion. (Left panel is redrawn with permission from *ECS Transactions* 77, 1155–1162 (2017))^13^.

Vineyard’s convolution approximation^16,17^,

6 $${G}_{{\rm{d}}}(r,t)\approx \int {{\rm{d}}}^{3}r^{\prime} g(r^{\prime} ){G}_{{\rm{s}}}(|\overrightarrow{r}-\overrightarrow{r}^{\prime} |,t),$$

provides an initial characterization for the *distinct* part of the Li^+^-Li^+^ van Hove function (Fig. 7, left panel redrawn from ref.^13^). Here, the convolution of the radial distribution function is made with the *self* part of the van Hove function that is generated by dynamics of Li^+^ in EDC. This approximation is consistent with the idea that *G*_d_(*r*, *t*) is a dynamical counterpart to *g*(*r*), the radial distribution function. The non-zero population in the core region surrounding *r* ≈ 0 describes correlation of Li^+^ jumps; that is, refilling a hole left by a Li^+^ ion with a neighboring Li^+^ ion.

### Vibrational power spectra of Li^+^

**Figure 8:**
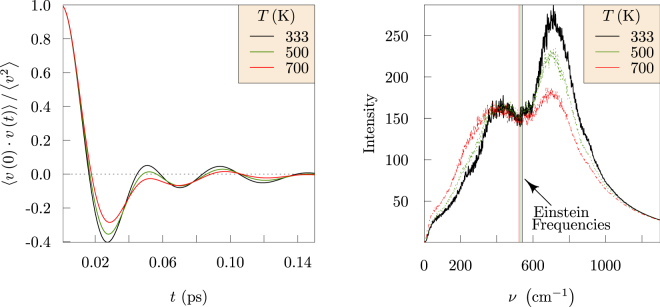
Velocity auto-correlation functions (left) for Li^+^ in EDC at different temperatures. Power spectra (right) for Li^+^ in EDC. Vertical lines near 570 cm^−1^ are Einstein frequencies (Eq. 7) of these ionic motions. The power spectra identify two prominent vibrational bands, near 400 cm^−1^ and 700 cm^−1^. The intensities of the high frequency modes diminish with increasing temperature.

**Figure 9:**
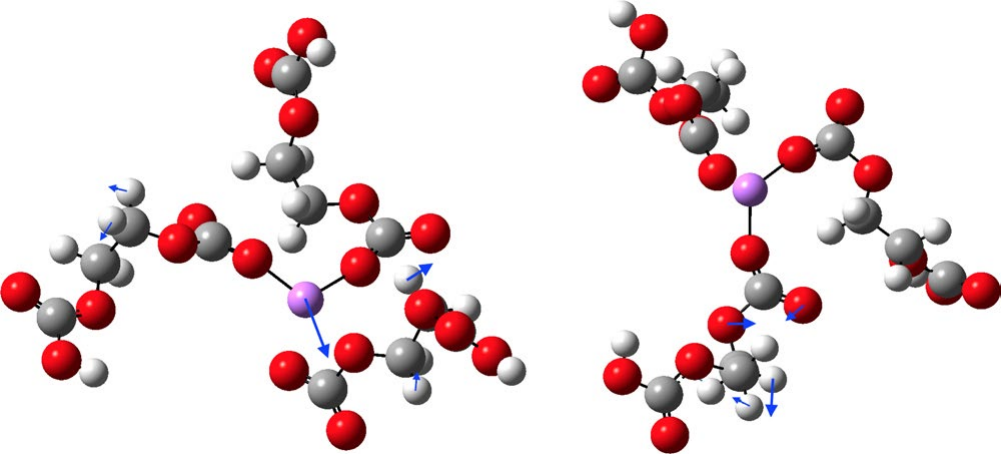
Vibrational modes due to prominent Li^+^ movement (left 436 cm^−1^) and solvent motion around 700 cm^−1^ (right). Li^+^ is surrounded by 3 EDC molecules. Adjacent carbonyl and ether oxygen of the same EDC molecule interact with Li^+^ (left). The Gaussian09^24^ software was used for these calculations with the b3lyp exchange-correlation density functional and 6–31 + g (d, p) basis set. The structures were sampled from the classical MD simulations and then the carboxyl groups not coordinating with the Li^+^ ions were neutralized by adding hydrogen atoms. The blue arrows indicate the atomic displacements for these normal mode frequencies^25^.

The vibrational power spectra are obtained by spectral decomposition of the velocity autocorrelation (Fig. 8, left). Since we are interested in Li^+^ transport, this analysis is carried out for Li^+^ atoms exclusively. This fact distinguishes our results from the FTIR spectrum of EDC molecule that was reported previously^2^. The spectral distribution for Li^+^ in EDC (Fig. 8, right) is bi-modal, with a temperature dependence at the higher frequency mode. Electronic structure calculations, using structures sampled from MD trajectories, confirm these modes and provide a molecular assignment (Fig. 9). The lower frequency mode (near 400 cm^−1^) corresponds to motion of a Li^+^ ion trapped in a cage formed by its nearest neighbors. The higher frequency mode (near 700 cm^−1^) corresponds to Li^+^ ion picking up the scissoring motion of a neighboring carbonate group.

The Einstein frequency (*ν*_e_) is obtained as the coefficient in the quadratic approximation to the velocity autocorrelation function at short times,

7 $$\langle \overrightarrow{v}(t)\cdot \overrightarrow{v}(0)\rangle /\langle {v}^{2}\rangle \approx 1-{({\nu }_{{\rm{e}}}t)}^{2}$$

In a simple Einstein model, all atoms vibrate with a single frequency. Fittingly, for the bi-modal spectra, *ν*_e_ lies in-between the two significant modes.

## Conclusions

Non-polarizable, classical force field parameters were used to study transport characteristics of Li^+^ in a model SEI layer composed of EDC. The structural results in EDC are consistent with prior studies that use polarizable force fields. An advantage of non-polarizable force fields is their ready availability in standard simulation packages and accessibility to MD studies over microsecond timescales. Thus, the dynamical characteristics presented here lay a basis for careful molecular-scale examination of the mechanism of transport of Li^+^ ions in the SEI.

These observations over microsecond simulation times provide new physical insights. Specifically, the results compare the glassy behavior of the ethylene dicarbonate SEI matrix with the fluid behavior of liquid ethylene carbonate (Fig. 6). Further, the Li^+^ MSDs examined in the nanosecond time intervals distinguish Li^+^ ion trapping in cages formed by the EDC matrix. Our results establish the sizes of the cages and the trapping lifetimes (Fig. 3), and also the dynamical motions of the Li^+^ ions when trapped (Fig. 8). The vibrational frequency of a trapped ion (about 440 cm^−1^) is confirmed by electronic structure calculations (Fig. 9). Our results invalidate a naive Einstein model of trapped ions that would be plausible otherwise. The van Hove correlation functions (Figs 4 and 7) provide information for analysis of the correlation of Li^+^ jumps.

## Methods

Li_2_EDC (Fig. 1) is known to be a dominant component of the SEI layer in lithium ion batteries involving carbonate solvents. Although Li_2_EDC is synthesized in crystalline form, its structure at the SEI layer is unknown^2,10^. We constructed a system of 256 Li_2_EDC moieties for our initial SEI studies. This system size is identical to previous molecular simulations performed using polarizable force fields^10,11^. For comparison, we also simulated a single Li^+^ ion solvated by 249 EC (Fig. 1). The GROMACS molecular dynamics simulation package^18^ was used for all simulations, and the necessary topology files for EDC and EC were created using non-polarizable all-atom optimized potentials for liquid simulations (OPLS-AA) force field parameters^19^. The partial charges on EC atoms were adjusted down to 80% to match experimental transport properties for EC^20^.

The EDC and Li^+^ ions were randomly placed into the simulation cell and MD simulations were performed at 700, 500 and 333 K. Since EDC ions are sluggish, configurations from the highest temperature calculation were used to obtain starting points for further simulations, cooled down to 500 K and subsequently to 333 K to study moderate temperature phenomena. Thus, the results presented here are based on amorphous configurations of the EDC/SEI layer. Although it is unclear that Li_2_EDC is crystalline at the SEI layer, we have simulated ordered layers and found that the solvent density and radial distribution functions are not substantially changed compared with amorphous Li_2_EDC. The ordered Li_2_EDC is more conductive compared to amorphous Li_2_EDC^11^, but formation of an ordered SEI structure is unlikely. Therefore, we here discuss only results of amorphous Li_2_EDC.

Periodic boundary conditions mimic the bulk environment in these calculations. A Nose-Hoover thermostat^21,22^ and a Parrinello-Rahman^23^ barostat were utilized to achieve equilibration in the *NpT* ensemble at 1 atm pressure. A 200 ns production run at 700 K was carried out after initial energy minimization and equilibration steps, then a 250 ns calculation at 500 K. Finally, a 1 *μ*s trajectory at 333 K temperature was constructed. Configurations were saved after each 1 ps of those production runs. A separate 1 ns simulation with a sampling rate of 1 fs was carried out at each temperature to calculate the time-independent pair correlation functions discussed below.

## Electronic supplementary material

Supplementary information
