# Supplementary material for: Molecular Dynamics of Lithium Ion Transport in a Model Solid Electrolyte Interphase
Source: Sci Rep. 2018 Jul 16;8:10736. doi: 10.1038/s41598-018-28869-x (PMC6048109; doi:10.1038/s41598-018-28869-x)
Supplement: Supplementary file 1 — Supplementary information [file 41598_2018_28869_MOESM1_ESM.pdf]

Molecular Dynamics of Lithium Ion Transport in a Model Solid Electrolyte Interphase

Ajay Muralidharan,[*,†] Mangesh I. Chaudhari,[*,‡] Lawrence R. Pratt,[*,†] and Susan B. Rempe[*,‡,¶]

†*Department of Chemical and Biomolecular Engineering, Tulane University, New Orleans, LA 70118, USA*

‡*Center for Biological and Engineering Sciences, Sandia National Laboratories, Albuquerque, NM 87185, USA*

¶*Corresponding author*

E-mail: amuralid@tulane.edu; michaud@sandia.gov; lpratt@tulane.edu; slrempe@sandia.gov

Supporting Information

Diffusion constant:

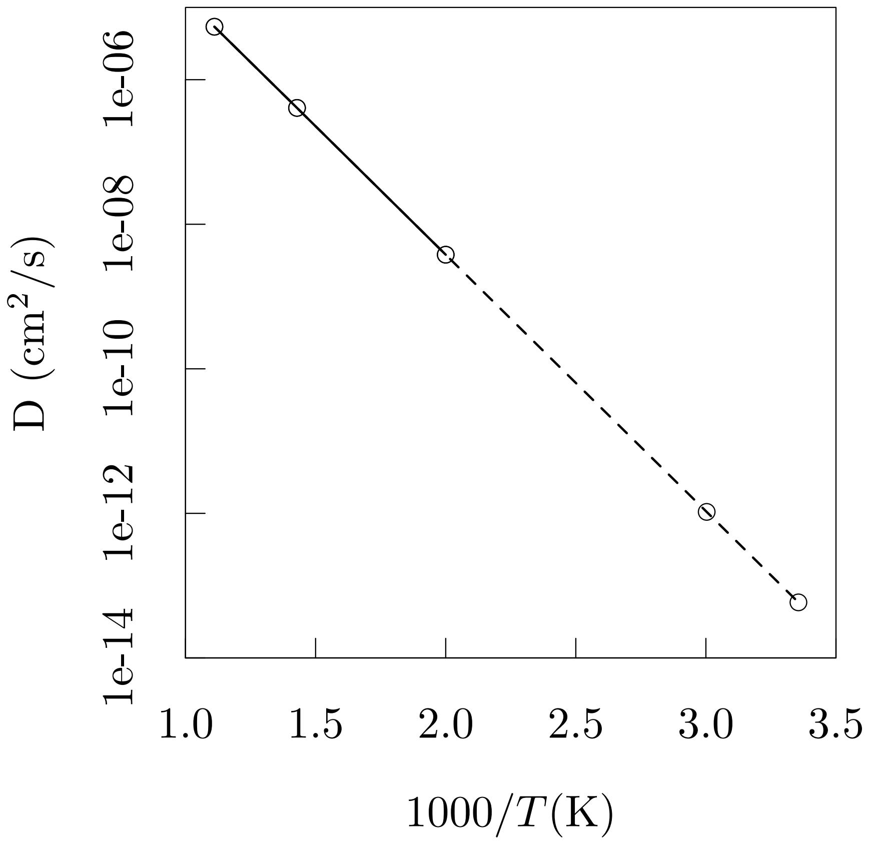

Figure 1: Arrhenius fit for the temperature dependence of Li^+ diffusivity. The low temperature values (10^{-12} cm^2/s at 333 K and 5.8 x 10^{-14} cm^2/s at 298 K) are obtained by extrapolation.

Conductivity:

At room temperature (298 K), the diffusion of EDC is insignificant compared to Li^+ and hence only Li^+ contributes to the conductivity. The conductivity (λ) given by the Nernst-Einstein relation, $\lambda = \frac{e^2}{V k_B T}$ ($n_{Li} D_{Li}$), is 4.5 x 10^{-9} S/cm. T, V, e, k_B, n_{Li}, D_{Li} stand for

temperature, volume of the simulation box, electron charge, Boltzmann constant, number of Li^+, and diffusivity of Li^+ respectively.

Force Field Parameters:

```
; ------------------------------------------------------------
; EDC topology for Solid Electrolyte Interphase simulations
; ------------------------------------------------------------
; This is a standalone topology file
; Note:
; This might be a non-standard force field location. When you use this topology, the
; force field must either be present in the current directory, or the location
; specified in the GMXLIB path variable or with the 'include' mdp file option.
;
[ moleculetype ]
; Name            nrexcl
EDC             3

[ atoms ]
;   nr       type  resnr residue  atom   cgnr     charge       mass  typeB    chargeB
        1  opls_465   1   EDC      C1     1   0.40600     12.011
        2  opls_466   1   EDC      O1     1   -0.58600     15.9994
        3  opls_466   1   EDC      O2     1   -0.58600     15.9994
        4  opls_467   1   EDC      O3     1   -0.35600     15.9994
        5  opls_468   1   EDC      C2     2   0.00800     12.011
        6  opls_140   1   EDC      H1     2   0.05700     1.008
        7  opls_140   1   EDC      H2     2   0.05700     1.008
        8  opls_468   1   EDC      C3     3   0.00800     12.011
```

```
     9  opls_140   1   EDC       H3    3   0.05700     1.008
    10  opls_140   1   EDC       H4    3   0.05700     1.008
    11  opls_467   1   EDC       O4    4   -0.35600     15.9994
    12  opls_465   1   EDC       C4    4   0.40600     12.011
    13  opls_466   1   EDC       O5    4   -0.58600     15.9994
    14  opls_466   1   EDC       O6    4   -0.58600     15.9994
```

```
[ bonds ]
1 2 1   0.127  476976.0
1 3 1   0.128  476976.0
1 4 1   0.148  179075.2
4 5 1   0.148  267776.0
5 6 1   0.110  284512.0
5 7 1   0.110  284512.0
5 8 1   0.153  224262.4
8 9 1   0.110  284512.0
8 10 1   0.110  284512.0
8 11 1   0.148  267776.0
11 12 1   0.148  179075.2
12 13 1   0.128  476976.0
12 14 1   0.127  476976.0
```

```
[ angles ]
1 4 5 1  117.162  694.544
2 1 3 1  131.306  200.000
2 1 4 1  112.723  694.544
3 1 4 1  115.971  694.544
```

4 5 6 1 110.294 292.880

4 5 7 1 110.284 292.880

4 5 8 1 106.028 418.400

5 8 9 1 111.075 313.800

5 8 10 1 111.050 313.800

5 8 11 1 106.028 418.400

6 5 7 1 108.123 276.144

6 5 8 1 111.050 313.800

7 5 8 1 111.075 313.800

8 11 12 1 117.162 694.544

9 8 10 1 108.123 276.144

9 8 11 1 110.284 292.880

10 8 11 1 110.294 292.880

11 12 13 1 115.971 694.544

11 12 14 1 112.723 694.544

13 12 14 1 131.306 200.000

[dihedrals]

2 1 4 5 3

3 1 4 5 3

1 4 5 6 3

1 4 5 7 3

1 4 5 8 3

4 5 8 9 3

6 5 8 9 3

7 5 8 9 3

4 5 8 10 3

6 5 8 10 3

7 5 8 10 3

4 5 8 11 3

6 5 8 11 3

7 5 8 11 3

5 8 11 12 3

9 8 11 12 3

10 8 11 12 3

8 11 12 13 3

8 11 12 14 3

[pairs]

5 2 1

5 3 1

6 1 1

7 1 1

8 1 1

9 4 1

9 6 1

9 7 1

10 4 1

10 6 1

10 7 1

11 4 1

11 6 1

11 7 1

12 5 1

```
12 9 1
12 10 1
13 8 1
14 8 1
; -------------------------------------------------------------
;  EC solvent topology for battery electrolytes
; -------------------------------------------------------------
;  Charges on EC atoms scaled to 80%
;  This is a standalone topology file
;
;  Note:
;  This might be a non-standard force field location. When you use this topology, the
;  force field must either be present in the current directory, or the location
;  specified in the GMXLIB path variable or with the 'include' mdp file option.
;

[ moleculetype ]
; Name            nrexcl
EC                  3

[ atoms ]
;   nr       type  resnr residue  atom   cgnr     charge       mass  typeB    chargeB
; residue   0 EC  rtp EC   q  0.0
     1   opls_774      0     EC     C3      1      0.0264   12.011   ; qtot 0.21
     2   opls_777      0     EC     H5      1      0.08328   1.008   ; qtot 0.24
     3   opls_777      0     EC     H6      1      0.08328   1.008   ; qtot 0.27
     4   opls_773      0     EC     O1      2     -0.37472 15.9994   ; qtot -0.18
```

```
    5   opls_772      0     EC     C4      2      0.87968  12.011   ; qtot 0.68
    6   opls_771      0     EC     O2      2     -0.51616 15.9994   ; qtot 0.18
    7   opls_773      0     EC     O3      2     -0.37472 15.9994   ; qtot -0.27
    8   opls_774      0     EC     C2      3      0.0264   12.011   ; qtot -0.11
    9   opls_777      0     EC     H4      3      0.08328   1.008   ; qtot -0.055
   10   opls_777      0     EC     H3      3      0.08328   1.008   ; qtot 0

[ bonds ]
;  ai    aj funct           c0            c1            c2            c3
    1     2     1
    1     3     1
    1     4     1
    1     8     1
    4     5     1
    5     6     1
    5     7     1
    7     8     1
    8     9     1
    8    10     1

[ pairs ]
;  ai    aj funct           c0            c1            c2            c3
    1     6     1
    2     5     1
    2     7     1
    2     9     1
    2    10     1
```

```
    3     5     1
    3     7     1
    3     9     1
    3    10     1
    4     9     1
    4    10     1
    5     9     1
    5    10     1
    6     8     1

[ angles ]
;  ai    aj    ak funct            c0            c1            c2            c3
    2     1     3     1
    2     1     4     1
    2     1     8     1
    3     1     4     1
    3     1     8     1
    4     1     8     1
    1     4     5     1
    4     5     6     1
    6     5     7     1
    5     7     8     1
    1     8     7     1
    1     8     9     1
    1     8    10     1
    7     8     9     1
    7     8    10     1
```

```
    9     8    10     1

[ dihedrals ]
;  ai    aj    ak    al funct            c0            c1            c2            c3
    2     1     4     5     3
    3     1     4     5     3
    8     1     4     5     3
    2     1     8     7     3
    2     1     8     9     3
    2     1     8    10     3
    3     1     8     7     3
    3     1     8     9     3
    3     1     8    10     3
    4     1     8     7     3
    4     1     8     9     3
    4     1     8    10     3
    1     4     5     6     3
    6     5     7     8     3
    5     7     8     1     3
    5     7     8     9     3
    5     7     8    10     3

; -------------------------------------------------------------
```
